# What have we learned about the processes involved in the Iowa Gambling Task from developmental studies?

**DOI:** 10.3389/fpsyg.2014.00915

**Published:** 2014-08-20

**Authors:** Mathieu Cassotti, Ania Aïte, Anaïs Osmont, Olivier Houdé, Grégoire Borst

**Affiliations:** ^1^Laboratory for the Psychology of Child Development and Education, CNRS Unit 8240, Sorbonne Paris Cité, Paris Descartes UniversityParis, France; ^2^Institut Universitaire de FranceParis, France

**Keywords:** Iowa Gambling Task, emotion-based learning, executive control, inhibition (psychology), children and adolescents, developmental psychology, loss aversion

## Abstract

Developmental studies using the Iowa Gambling Task (IGT) or child-friendly adaptations of the IGT converged in showing that children and adolescents exhibit a strong bias in favor of disadvantageous choices whereas adults learn to decide advantageously during the course of the task. In the present article, we reviewed developmental studies that used the IGT or child-friendly adaptations of the IGT to show how these findings provide a better understanding of the processes involved in decision-making under uncertainty. For instance, developmental studies have underlined that until late adolescence, the dominant strategy is to focus only on the frequency of punishment and to choose among options with infrequent losses. Indeed, school-aged children and adolescents' choices in the IGT seem to be guided by the loss frequency leading them to fail in distinguishing between advantageous and disadvantageous options. In addition, recent developmental studies revealed that adults switch less often after losses than school-aged children and adolescents. These findings suggest that psychological tolerance to loss may facilitate learning the characteristics of each option, which in turn improves the ability to choose advantageously. In conclusion, developmental studies help us refine our understanding of decision-making.

## Introduction

In most situations of everyday life, people make decisions in circumstances where some information about the potential outcomes of choices are lacking and must be inferred from experience. Over the last two decades, considerable efforts in the field of psychology and neuroscience have been leveled at identifying the processes involved in this category of decision-making situations (Bechara and Damasio, 2005; Dunn et al., 2006). In particular, from a developmental perspective, a growing body of research suggests that the ability to decide advantageously evolves with age (see Table 1; Crone et al., 2005; Cassotti et al., 2011; Aïte et al., 2012; Beitz et al., 2014). Therefore, in the present article, we reviewed these developmental studies to demonstrate how it helps us refine our understanding of decision-making under uncertainty.

**Table 1:** **Example of developmental studies on decision-making under ambiguity**.

**Authors**	***N***	**Ages**	**Task**	**Option A**	**Option B**	**Option C**	**Option D**	**Results**
Aïte et al., 2012	44	7–32	SGT (100)	Gain: 10–30(80%)	Gain: 10–30(80%)	Gain: 105(20%)	Gain: 55(20%)	• Children and adolescents were only guided by loss-frequency while adults managed to choose advantageously only when the frequency of loss was low.
				Loss: 105(20%)	Loss: 55(20%)	Loss: 10–30(80%)	Loss: 10–30(80%)	
				Net score: −50	Net score: +50	Net score: +50	Net score: −50	
								• Adults used more “win-stay” and “loss-stay” strategies. A negative correlation was observed between the proportion of “loss-stay” strategy and disadvantageous selection in option A.
Cassotti et al., 2011	84	9–28	IGT (100)	Gain: 8–12(100%)	Gain: 8–12(100%)	Gain: 4–6(100%)	Gain: 4–6(100%)	• Adults switched less often after gains and persevered more after loss compared to children and adolescents.
				Loss: 15–35(50%)	Loss: 125(10%)	Loss: 2–8(50%)	Loss: 25(10%)	
				Net score: −50	Net score: −50	Net score: +50	Net score: +50	
								• Children and adolescents focused on options with low loss-frequency.
Crone et al., 2005	140	7–15	HDT (150)	Gain: 4(100%)	Gain: 4(100%)	Gain: 2(100%)	Gain: 2(100%)	• Only the BD version revealed developmental differences with younger children (7–9 years old) failing to choose advantageously.
				Loss: 8–12(50%)	Loss: 50(10%)	Loss: 1–3(50%)	Loss: 10(10%)	
			AACC	Net score: −10	Net score: −10	Net score: +10	Net score: +10	
			AC	Gain: 4(100%)		Gain: 2(100%)		• The number of options available did not influence performances (i.e., no difference between AACC and AC).
				Loss: 8–12(50%)		Loss: 1–3(50%)		
				Net score: −10		Net score: +10		
			BD		Gain: 4(100%)		Gain: 2(100%)	• Participants used “win-stay” and “loss-shift” strategies.
					Loss: 50(10%)		Loss: 10(10%)	
					Net score: −10		Net score: +10	
Crone and van der Molen, 2004	331	6–25	HDT (200)	Gain: 4(100%)	Gain: 4(100%)	Gain: 2(100%)	Gain: 2(100%)	• Adults chose advantageously from the 3rd block.
				Loss: 8–12(50%)	Loss: 50(10%)	Loss: 1–3(50%)	Loss: 10(10%)	• Children failed to disengage from disadvantageous options.
				Net score: −10	Net score: −10	Net score: +10	Net score: +10	
								• Adolescents (13–15 years old) started to show a slight advantageous preference at the end of the task.
								• At every age there is a preference for low-loss frequency options.
Crone and van der Molen, 2007	81	8–18	HDT (100)	Gain: 4(100%)	Gain: 4(100%)	Gain: 2(100%)	Gain: 2(100%)	• Only adolescents (16–18 years old) showed an increase number of advantageous choices and larger anticipatory autonomic responses for frequent-loss options than for infrequent-loss options.
				Loss: 8–12(50%)	Loss: 50(10%)	Loss: 1–3(50%)	Loss: 10(10%)	
				Net score: −10	Net score: −10	Net score: +10	Net score: +10	
								• Similar autonomic responses (SCRs and HR) to gains and losses were observed for each age group.
van Duijvenvoorde et al., 2010	94	13–15	HDT (200)	Gain: 4(100%)	Gain: 4(100%)	Gain: 2(100%)	Gain: 2(100%)	• At the end of the task, adolescents focused on options with low loss-frequency (i.e., B and D).
				Loss: 8–12(50%)	Loss: 50(10%)	Loss: 1–3(50%)	Loss: 10(10%)	
				Net score: −10	Net score: −10	Net score: +10	Net score: +10	

*Each line refers to authors, age and number of participants, task design and major findings of the study*.

According to the Somatic Marker Hypothesis (SMH) (Bechara et al., 2000; Bechara and Damasio, 2005), emotion-related signals, developed from past experience of the emotional consequences following choices, guide decision making in situations of uncertainty. More specifically, this theory assumes that advantageous decisions rely on the development of an integral emotional reactivity (i.e., somatic maker). These somatic markers allow one to avoid disadvantageous options and to develop a preference for advantageous ones.

Most of the empirical supports for this model came from studies using the Iowa Gambling Task (IGT, Bechara and Damasio, 2005), a task initially developed to simulate the inherent uncertainty of daily-life decisions' situations through an opaque gain-loss schedule. The IGT consists of a card game in which participants are instructed to win as much money as possible by selecting among four possible decks of cards (labeled A, B, C, or D). Importantly, the decks' characteristics are not disclosed, and should be gradually inferred from feedbacks obtained during the game. Indeed, feedbacks are provided after each selection so that participants systematically win some money, but also and unforeseeably lose some. The four decks differ in the magnitude of wins and losses in such a way that to succeed at this game, players must withdraw from attractive but disadvantageous in the long term decks (A, B) and opt for less attractive but advantageous in the long term decks (C, D).

Typically, healthy adults progressively and implicitly learn to choose advantageously during the course of the task (Crone and van der Molen, 2004; Dunn et al., 2006; Cassotti and Moutier, 2010; Cassotti et al., 2011; Turnbull et al., 2014). In addition, advantageous performances has been linked to an anticipated emotional reactivity, as measured by Skin Conductance Responses (SCRs). Indeed, healthy participants display gradually higher anticipatory SCRs before picking a card in disadvantageous decks than in advantageous ones, suggesting that an emotional warning signal leads them to avoid disadvantageous choices (Bechara et al., 2000; Carter and Smith-Pasqualini, 2004; Guillaume et al., 2009). Similarly, anticipatory heart rate responses and SCRs were found to be critical to distinguish good and bad performers in such decision-making under uncertainty tasks suggesting a key role of this anticipated emotional reactivity in the ability to make advantageous decisions (Crone et al., 2004; Denburg et al., 2006).

In agreement with the SMH both neuropsychological studies and neuroimaging studies have shown the involvement of specific brain regions implicated in emotional processes in the IGT (Reimann and Bechara, 2010). More specifically, poor IGT performance and lower anticipatory SCRs were observed in amygdala-damaged patients (Bechara et al., 1999) as well as in patients with ventromedial prefrontal cortex (VMPFC) lesions (Bechara and Damasio, 2005). Using functional Magnetic Resonance Imaging, several studies have reported the activation of a prefrontal network including the VMPFC in the IGT which provides convergent evidence that decision-making under uncertainty might rely on an emotional neural circuitry (Lawrence et al., 2008; Li et al., 2010).

Additional empirical evidences in favor of the SMH came from studies exploring the influence of the emotional context on advantageous decisions in the IGT (Hinson et al., 2006; Davies and Turnbull, 2011; Aïte et al., 2013; see Turnbull et al., 2014 for a review). In response to remaining critics about the reliability of electrophysiological measures such as SCRs (Tomb et al., 2002; Dunn et al., 2006), Aïte et al. (2013) recently designed an emotional priming paradigm of the IGT to determine whether the ability to choose advantageously in ambiguous situations is driven by an integral emotional signal as assumed by the SMH. In this study, the emotional context was either congruent or incongruent with the feedback delivered after each choice and was manipulated using pictures of either happy or fearful faces (Tottenham et al., 2009). The results of this study strongly support the SMH by evidencing that decision making was improved when the integral emotional signal was reinforced by a congruent emotional context and impaired when the integral emotional signal was disrupted by an incongruent emotional context.

## Child friendly adaptation of the IGT and advantageous decision-making

Given that neuroimaging studies over the past 20 years have consistently shown continuing neuroanatomical and neurofunctional development of the prefrontal cortex across childhood and adolescence (Crone and Dahl, 2012), numerous developmental studies examining decision making under uncertainty have focused on changes in performance on child friendly adaptations of the IGT between school-aged children and adolescence (Crone et al., 2005; Cassotti et al., 2011; Beitz et al., 2014).

One of the first studies that investigated developmental changes in decision-making ability during adolescence showed that this ability continues to improve until late adolescence and even in adulthood (Crone and van der Molen, 2004). The authors of this study have designed an age-appropriate version of the IGT: the Hungry Donkey Task (HDT), in which the stimulus presentation was modified to make the task more meaningful for children (Crone and van der Molen, 2004). Indeed, rather than picking cards to win money for themselves, participants are invited to assist a hungry donkey in winning as many apples as possible by opening doors. Two doors (A and B) constitute disadvantageous choices (resulting in overall loss), and two doors (C and D) advantageous choices (resulting in overall gain). Critically, this task maintains the basic format and a similar schedule of rewards and losses as those described by Bechara et al. (1994). As in other IGT studies, participants' performance is measured in terms of changes in individuals' net scores for blocks of 20 trials by subtracting the number of choices in disadvantageous doors from the number choices in advantageous doors.

In a series of behavioral studies, Crone and colleagues have demonstrated that 6- to 9-year-old children and 10- to 12-year-old children fail to avoid disadvantageous options during the course of the task, as opposed to 13- to 15 year-old adolescents, who gradually learn to choose advantageously (Crone and van der Molen, 2004, 2007; Crone et al., 2005; Huizenga et al., 2007). Nevertheless, adolescents' performance is still suboptimal compared to adults, suggesting that the ability to distinguish between advantageous and disadvantageous options continues to improve during late adolescence (Overman, 2004; Overman and Pierce, 2013). Although some authors point out that there may be differences between the development of decision making involving personal gain or loss and decision making that leads to help another such as in the HDT, developmental studies using the standard IGT have confirmed that advantageous decision making progressively increases until early adulthood (Hooper et al., 2004; Cassotti et al., 2011; Beitz et al., 2014). It has been proposed that this age-related improvement in performance on child adaptations of the IGT during adolescence may be due the slow functional maturation of the VMPFC until early adulthood (Crone and van der Molen, 2004, 2007).

## Development of the frequency bias

Because the IGT is a complex task, different factors may contribute to the similar decision-making deficit observed in children and VMPFC patients. In support of this hypothesis, developmental studies revealed that children have a marked preference for options associated with infrequent losses, regardless of whether these options are advantageous or disadvantageous in the long run (Crone and van der Molen, 2004, 2007; Crone et al., 2005; Huizenga et al., 2007; Carlson et al., 2009; Cassotti et al., 2011). Indeed, the four options proposed in the IGT and the child-friendly versions of this task also differ in the frequency of losses, with two options associated with a low frequency of losses (10% for decks/doors B and D) and two options associated with a medium frequency of losses (50% for decks/doors A and C). Developmental studies converged in showing that children and adolescents increasingly opt for choices associated with infrequent, rather than frequent losses during the task and that this frequency bias decreases with age (Huizenga et al., 2007). In contrast, adults display a strong preference for low loss frequency choices early in the task but progressively opt for advantageous options during the course of the task. To the best of our knowledge, this specific sensitivity to options associated with a low frequency of losses has not been observed in VMPFC patients.

Furthermore, Crone and van der Molen (2007) demonstrated that adolescents display higher anticipatory SCRs preceding a choice among options associated with frequent losses in contrast to adults who generate progressively higher anticipatory SCRs before disadvantageous selections (Bechara and Damasio, 2005). These results suggest (a) that loss frequency highly influences children and adolescents' decision making, and (b) that children and adolescents exhibit difficulties in considering both the frequency and the amount of loss leading them to make long-term disadvantageous decisions (see also Jansen et al., 2011; van Duijvenvoorde et al., 2012). Interpreted within the framework of the SMH, these data suggest that adolescents reactivate the negative emotional responses associated with high-loss frequency options without taking into account the final outcome.

Interestingly, recent evidence underlined a comparable inability to integrate both loss frequency and final outcome in adults' decision making (Lin et al., 2009; Cassotti and Moutier, 2010). For example, using the Soochow Gambling Task (SGT), an adaptation of the IGT designed to directly contrast the impact of loss frequency and final outcome on decision making, Lin et al. (2009) demonstrated that adults substantially based their choices on loss frequency rather than on final outcome (i.e., the advantageous or disadvantageous nature of the decks on the long run). More specifically, when the frequency of loss in the decks is manipulated to take either one of two extreme values (80 vs. 20%), adults are guided by gain–loss frequency leading them to fail in distinguishing between advantageous and disadvantageous choices. Given that this loss-frequency bias was observed only in children and adolescents when the classical IGT was used, Aïte et al. (2012) further explored developmental changes in the ability to consider both the loss frequency and the final outcome in decision making using an age adapted version of the SGT. Results confirmed that children and adolescent not only preferred choices associated with infrequent losses in the SGT but also failed to differentiate advantageous options from disadvantageous ones, a developmental pattern similar to the one previously evidenced using the IGT. Contrarily to Lin et al.'s study (2009), findings indicated that adults did manage to consider the final outcome when making their decision but only for options associated with low-loss frequency. Thus, adults are not only guided by the loss frequency but also by the amount of the loss as evidenced by a preference for the advantageous option among the low-loss frequency options. Taken together, developmental studies using the IGT, the HDT, or the SGT converge in showing a robust frequency bias in childhood and adolescent that decrease in adults (Crone et al., 2005; Crone and van der Molen, 2007; Aïte et al., 2012).

## Strategic adjustments of decision making

A large majority of previous studies measured performance in the IGT or child adaptations of the IGT in terms of changes in difference between the number of advantageous selections and the number of disadvantageous choices. However, such measures provide no information on the strategic adjustments that immediately follow gains and losses over the course of the task. Given that participants can chose any of the four options on each trial, age differences observed in the standard net score and the tendency to focus on loss frequency could result from age differences in the strategic exploration of the four options.

In line with this hypothesis, Cassotti et al. (2011) have recently examined response-switching behavior following rewards and punishments in children, adolescents and adults. In this study, adults tended to persevere with the same choice after a win (i.e., a “win–stay” strategy) and to shift to a new choice after a loss (i.e., a “loss–shift” strategy). In contrast, children and adolescents failed to control a spontaneous tendency to explore the different options as shown by a higher frequency of switches following gains and losses in children and adolescents than in adults. Another study has not only confirmed these developmental differences but has also outlined a possible role for these strategic adjustments following gains and losses (Aïte et al., 2012). Given that the proportion of switches after losses correlated negatively with the number of advantageous selections, the reduced loss-shift pattern of response observed in adults, as compared to the two younger groups, could constitute a critical adaptive process allowing one to choose advantageously. Indeed, adults' tolerance to loss may allow them to learn the characteristics of each option and to increase their ability to consider not only loss frequency but also final outcome.

In line with these developmental studies (Cassotti et al., 2011; Aïte et al., 2012; van Duijvenvoorde et al., 2012) computational models that have included win-stay/loss-shift strategies to predict performance in the IGT (Worthy et al., 2007; Worthy and Maddox, 2012) converged in showing that the tendency to inhibit the loss-shift response is a central component of decision-making behavior in the IGT. Altogether, these results suggest that immature decision making may be due to the difficulty to execute inhibitory control on an automatic loss-shift response.

## Conclusion

In the present review, we have discussed how developmental studies have made significant progress in the understanding of the processes involved in emotional-based learning in the IGT. Behavioral and electrophysiological studies have clearly demonstrated a focus on loss-frequency (Crone et al., 2005; Crone and van der Molen, 2007) and a preponderance of a loss-shift strategy in children and adolescent as compared to adults (Aïte et al., 2012). In line with recent behavioral and neuroimaging studies (Spear, 2011; Habib et al., in press) children and adolescent might be more focused on loss frequency and might rely more on a loss-shift strategy because of an exacerbate aversion to losses. Given that only adults have the ability to choose advantageously (Crone and van der Molen, 2004, 2007; Overman, 2004; Overman and Pierce, 2013), the subtle developmental differences regarding the factors that guide decision making and the strategic adjustment that are used might reflect the critical components needed for making advantageous decisions. Indeed, the data collected in developmental studies suggest that the ability to develop emotion-related signals that integrate both loss-frequency and final outcome requires inhibitory control of intuitive exploration strategies. As such, the present article provides new fuel for the current debates on the respective contribution of executive control and emotion-based learning in the IGT.

### Conflict of interest statement

The authors declare that the research was conducted in the absence of any commercial or financial relationships that could be construed as a potential conflict of interest.
